# Severe consequences of uncontrolled tertiary hyperparathyroidism in a limited resources setting: a case report

**DOI:** 10.3389/fneph.2025.1645622

**Published:** 2025-09-01

**Authors:** Beatriz Mejía Raudales, Carlos Andres Portillo Muñoz, Genesis Sarahi Chavez, Simmons Gough

**Affiliations:** ^1^ Universidad Catolica de Honduras, Tegucigalpa, Honduras; ^2^ Universidad Nacional Autonoma de Honduras, Tegucigalpa, Honduras; ^3^ Department of Radiology, Hospital Civil de Guadalajara, Unidad Hospitalaria Fray Antonio Alcalde, Guadalajara, Mexico

**Keywords:** chronic kidney disease, parathyroid gland, secondary hyperparathyroidism, renal osteodystrophy, hemodialysis

## Abstract

Tertiary hyperparathyroidism (THPT) is a severe complication of uncontrolled secondary hyperparathyroidism, typically associated with advanced-stage chronic kidney disease (CKD). We present the case of a Honduran patient with a long-standing history of CKD secondary to severe preeclampsia, who developed THPT following the discontinuation of her treatment due to financial constraints and the COVID-19 pandemic. The patient exhibited severe maxillofacial deformities, functional limitations, and widespread skeletal abnormalities. Despite initial management with medications such as paricalcitol and alfacalcidol, the lack of access to appropriate therapies and the postponement of a planned parathyroidectomy worsened her condition. This case highlights the importance of early diagnosis and timely intervention, particularly in resource-limited settings, emphasizing the urgent need for kidney transplant programs and improved preventive strategies in developing countries.

## Introduction

Tertiary hyperparathyroidism (THPT) is a serious consequence of uncontrolled secondary hyperparathyroidism, typically observed in patients with advanced stages of chronic kidney disease (CKD). This condition results from prolonged stimulation of the parathyroid glands, leading to their autonomous overactivity and resistance to normal regulatory mechanisms, such as serum calcium and active vitamin D. Without proper management, THPT can cause significant complications, including severe mineral-bone disorders and extra-skeletal manifestations (1, 2).

Globally, the prevalence of CKD is estimated to range from 7% to 12%, with regional variations influenced by access to medical care and early diagnostic practices. In developing countries like Honduras, the lack of standardized protocols and the burden on public health systems complicate timely diagnosis and management of the disease. In advanced stages of CKD, more than 80% of patients with a glomerular filtration rate below 20 ml/min/1.73 m² show elevated parathyroid hormone (PTH) levels, and up to 54% of end-stage renal disease patients present with some degree of hyperparathyroidism, potentially progressing to THPT if inadequately treated (3–6).

The management of hyperparathyroidism in CKD, according to KDIGO guidelines, involves the use of calcimimetics, calcitriol, or vitamin D analogs, either as monotherapy or in combination. However, in severe cases such as THPT, parathyroidectomy is considered the definitive treatment if pharmacological therapies fail (7).

This case report discusses the severe outcomes of uncontrolled THPT in a patient with advanced CKD, highlighting therapeutic challenges in a resource-limited setting and the clinical reality faced in developing countries. The report follows the CARE guidelines for case report preparation (8).

## Case report

A 48-year-old Honduran mestizo woman was admitted with a 19-year history of CKD, secondary to severe preeclampsia during her fourth pregnancy. Since then, she has been on renal replacement therapy via hemodialysis and has had secondary arterial hypertension. In 2019, the patient noticed a non-tender, immobile, solid mass on the anterior half of the hard palate, prompting her to seek medical consultation. The primary care physician diagnosed secondary hyperparathyroidism and referred her to the nephrology service, where treatment with paricalcitol was initiated, but later discontinued due to financial constraints. An alternative therapy with alfacalcidol was also unsuccessful. Consequently, parathyroidectomy was planned for the same year. However, the onset of the COVID-19 pandemic and the subsequent strain on public health systems prevented the procedure, and the patient lost routine hospital follow-up **Table 1**.

**Table 1:** Timeline.

Date/year	Event
2005–2006	Onset of chronic kidney disease (CKD) following severe preeclampsia during 4th pregnancy; patient starts hemodialysis.
2019	Patient notices a non-tender, immobile, solid mass on the hard palate; diagnosed with secondary hyperparathyroidism.
2019	Treatment initiated with paricalcitol but later discontinued due to financial constraints. Alfacalcidol tried without success.
2019	Parathyroidectomy planned, but cancelled due to COVID-19 pandemic and health system strain. Patient loses follow-up.
2019–2024	No treatment received for hyperparathyroidism. Progression to tertiary hyperparathyroidism.
2024	Patient presents with severe maxillofacial deformities, thoracic and limb deformities, ulcerated oral mucosa, functional disability.
2024	Labs: PTH >2500 pg/ml; Vitamin D 17.21 ng/ml; Phosphorus 4.6 mg/dl; Calcium 8.09 mg/dl; Total proteins 6.4 g/dl (Albumin 2.2, Globulins 4.2). Radiologic evidence of skeletal deformities.
2024	Dialysis parameters adjusted; parathyroidectomy rescheduled. Patient resumes medical care. Post OP care planned.
2025	Patient’s death due to Cardiovascular complications at home

**Figure 1:**
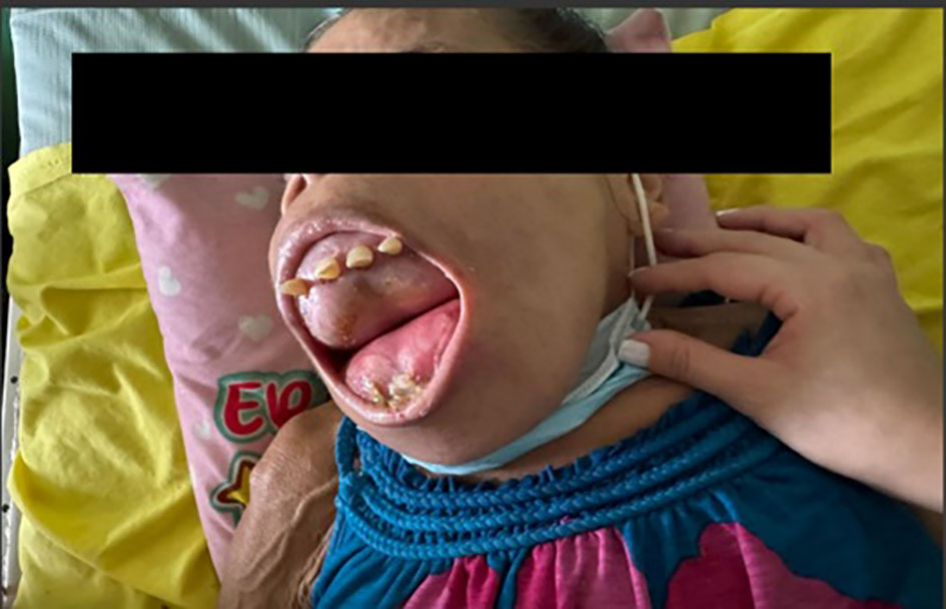
Facial anatomical changes experienced by the patient associated with tertiary hyperparathyroidism. There is marked maxillofacial deformity, preventing mouth closure and resulting in the subsequent exposure of the oral mucosa.

**Figure 2:**
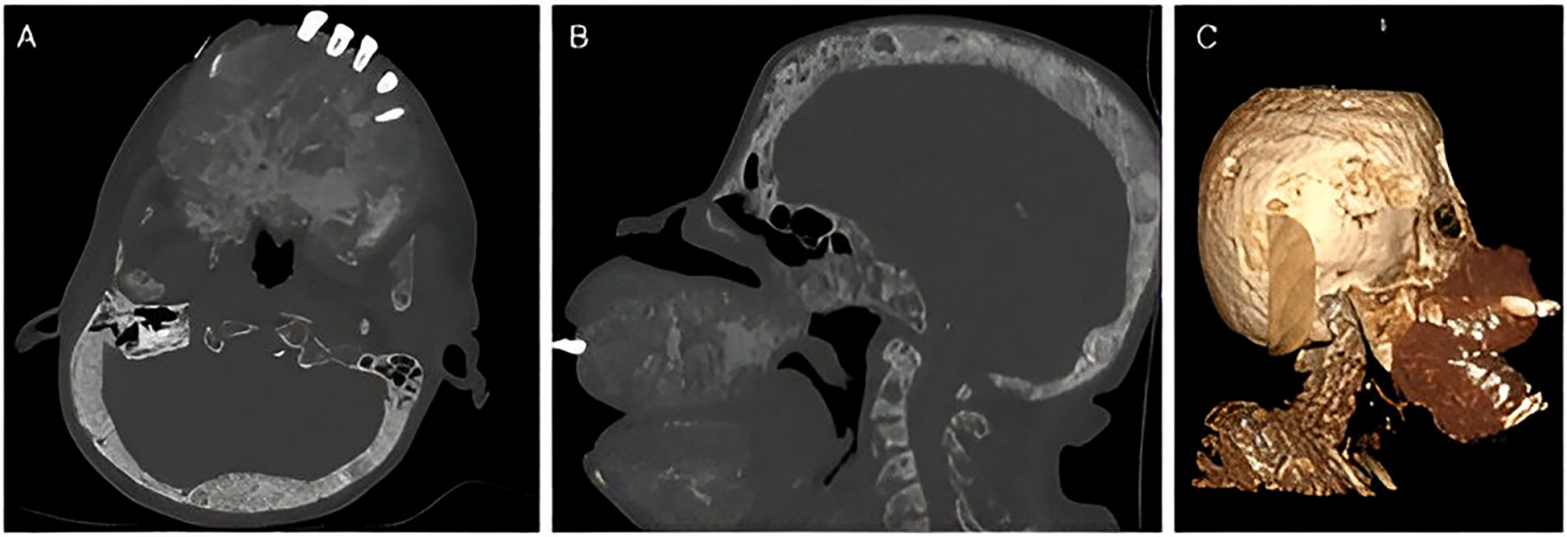
Non-contrast axial computed tomography in bone window at the level of the hard palate and skull base reveals an expansive, permeative osteoclastic lesion that distorts the architecture of the facial skeleton, findings suggestive of an extensive brown tumor. Additionally, a permeative appearance is noted in both the occipital and temporal bones **(A)**. Sagittal reconstruction in the same window demonstrates the previously mentioned changes, confirming their persistence in the rest of the cranial bones, mandible, and cervical spine **(B)**. Volumetric reconstruction illustrates the previously described bone deformity **(C)**.

Over the following five years, the patient did not receive any medical treatment for her secondary hyperparathyroidism reaching 2024 with extensive maxillary and mandibular deformities, affecting the entire oral vestibule, preventing mouth closure, proper oral hygiene, speech, and dental care, along with multiple areas of ulceration and bleeding in the friable and exposed oral mucosa. **Figure 1**. Craniofacial CT imaging showed an extensive osteolytic lesion involving the hard palate and skull base, consistent with a brown tumor, with additional bone destruction noted in the occipital, temporal, mandibular, and cervical regions **Figure 2**.

**Figure 3:**
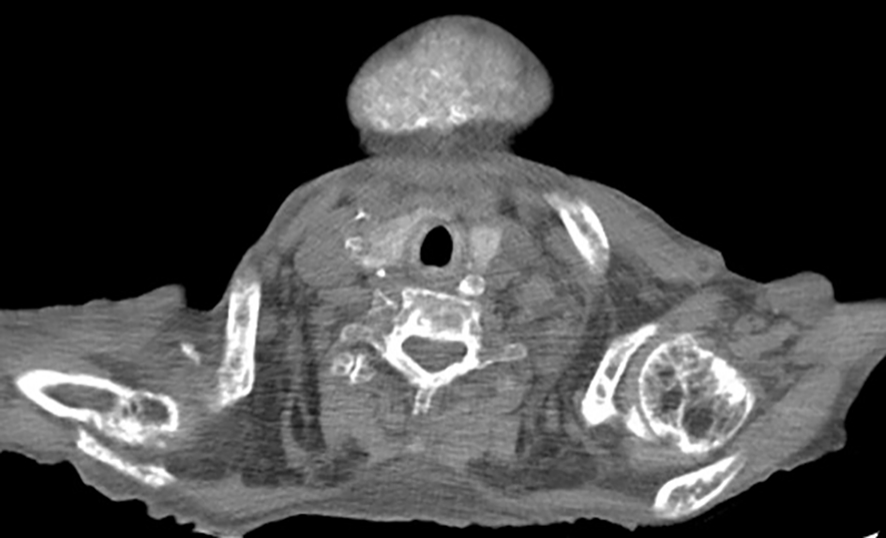
Non-contrast axial computed tomography of the lower neck in soft tissue window shows multiple ovoid nodular lesions in the thyroid gland, which are isodense to muscle tissue. Additionally, calcifications are observed along the course of both carotid arteries, suggestive of calcified atheromatous plaques.

**Figure 4:**
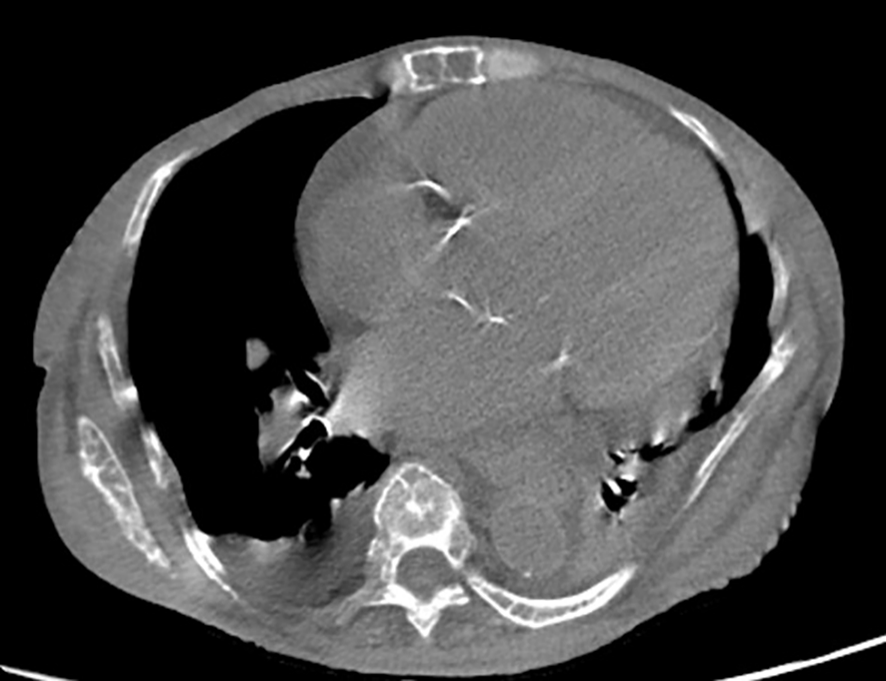
Non-contrast axial computed tomography in a soft tissue window at the level of the cardiac base reveals calcifications in the region of the heart valves. Incidentally, bilateral pleural effusion and passive atelectasis are observed.

Radiological findings confirmed a significant reduction in bone mineral density in the limbs, consistent with advanced renal osteodystrophy, along with expansive bone deformities affecting the thoracic cage and skull. These findings reflect both generalized osteopenia and localized bone remodeling associated with tertiary hyperparathyroidism. CT imaging of the neck in soft tissue window identified multiple ovoid nodular lesions within the thyroid gland isodense to muscle, along with vascular calcifications along the carotid artery pathways, indicative of atherosclerotic involvement. **Figure 3**. Furthermore, axial CT imaging at the level of the cardiac base revealed calcifications in the heart valve region, accompanied by bilateral pleural effusion and passive atelectasis **Figure 4**.

Laboratory findings revealed PTH levels >2500 pg/ml, vitamin D at 17.21 ng/ml, phosphorus at 4.6 mg/dl, calcium at 8.09 mg/dl, total proteins at 6.4 g/dl (albumin 2.2 g/dl, globulins 4.20 g/dl), and TSH at 1.62 μg/ml **Table 2**. Dialysis parameters were adjusted to resolve the acute dialysis needs, and surgical intervention was rescheduled, considering the risk-benefit ratio and the patient’s wish to pursue surgery to prevent further deterioration.

**Table 2:** Laboratory values on admissions.

Blood tests	Patient admission value	Reference range
Intact Parathyroid Hormone	>2500 pg/mL	3–2500 pg/mL
TSH	1.62 µIU/mL	0.4-4.0 µIU/mL
Vitamin D	17.21 ng/ml	>30 ng/mL
Calcium, non-ionized	8.09 mg/dl	8.60-10.00 mg/dl
Phosphorus	4.6 mg/dl	2.5-4.9 mg/dL
Total protein	6.4 g/dl	6.4-8.2 g/dl
Albumin	2.2 g/dL	3.4 – 5.0 g/dL
Globulins	4.20 g/dL	2.0 – 4.20 g/dL
Serum Creatinine	3.72 mg/dL	0.50 – 1.10 mg/dL

In the meantime, the patient remained under regular nephrology follow-up, including monthly laboratory assessments. Postoperative evaluations had also been arranged with maxillofacial surgery to explore therapeutic options for her craniofacial abnormalities, and with endocrinology to monitor for potential complications such as postoperative hypoparathyroidism. Unfortunately, before the planned intervention could take place, the patient passed away due to cardiovascular complications related to her underlying disease at home.

## Discussion

Tertiary hyperparathyroidism (THPT) represents the most advanced and least common stage of hyperparathyroidism. In contrast to primary hyperparathyroidism (PHPT), which affects between 0.1% and 1% of the general population and is caused by intrinsic dysfunction of the parathyroid glands, THPT develops after years of uncontrolled secondary hyperparathyroidism (SHP). SHP is a physiological response to hypocalcemia and vitamin D deficiency in CKD, affecting 10% to 20% of these patients, with 5% experiencing the severe form of the disease (9–13).

The primary causative factors include early phosphate retention, subsequent elevation of fibroblast growth factor 23 (FGF-23), inhibition of the CYP27B1 enzyme involved in calcitriol synthesis, and decreased expression of calcium-sensing receptors (CaSR) in parathyroid cells. These factors, coupled with a decrease in the total number of functional nephrons, lead to reduced levels of active vitamin D (calcitriol), diminishing its inhibitory effect on PTH secretion. Initially, PTH secretion is regulated by calcium levels, but it eventually becomes independent, resulting in tertiary hyperparathyroidism, where PTH release is autonomous, and no longer dependent on serum calcium levels (2).

In this case, progression to THPT was due to inadequate management of secondary hyperparathyroidism, compounded by limited access to effective treatments such as calcimimetics and vitamin D analogs. KDIGO guidelines advocate for the early use of these agents to control PTH levels in CKD and prevent the development of skeletal and extra-skeletal complications. However, in resource-limited environments, such as many developing countries, these therapies are often inaccessible or discontinued due to economic constraints, as occurred with this patient (4, 7).

Studies, such as that by Can et al. (14), have documented similar THPT cases with prominent maxillofacial involvement. Unlike the present case, their patient underwent kidney transplantation, which, although not always resolving tertiary hyperparathyroidism, significantly improves the prognosis. In the context of Honduras, the absence of a national kidney transplant program exacerbates the prognosis for patients who remain on prolonged dialysis, increasing the likelihood of THPT progression (9, 15).

THPT exerts a significant burden on patients with CKD, affecting both skeletal integrity and overall health status. In advanced stages, especially when craniofacial involvement occurs, patients may develop serious structural and functional complications. These can include compromised upper airway patency, impaired vision and hearing, difficulties with the oral phase of swallowing, and various neurological and psychiatric manifestations, as outlined by Sabanis et al. in their review of uremic leontiasis osse (7).

This case illustrates the disparity in the management of CKD and THPT between developed and developing countries. While patients in wealthier nations have access to kidney transplantation and advanced treatments, economic barriers and overwhelmed healthcare systems in regions like Honduras hinder effective management. As a result, patients who cannot adequately control their secondary hyperparathyroidism progress to more severe and resistant forms of the disease (4).

Despite these challenges, the patient eventually resumed medical care; however, she sadly passed away due to cardiovascular complications while awaiting parathyroidectomy. This fatidic outcome underscores the urgent need to reinforce CKD management programs in low-resource settings, with a strong emphasis on early detection and treatment of hyperparathyroidism to prevent irreversible complications. In cases of tertiary hyperparathyroidism, especially those presenting with severe craniofacial involvement, bone deformities often persist despite appropriate interventions such as parathyroidectomy or even renal transplantation (16). Once advanced skeletal disfigurement has occurred, reversal is unlikely, and patients frequently require additional reconstructive procedures, including oral and maxillofacial surgery.

In patients with CKD, especially those undergoing dialysis and diagnosed with secondary or tertiary hyperparathyroidism, hypoparathyroidism is a known postoperative complication following parathyroidectomy. This condition arises from inadequate parathyroid hormone (PTH) production after surgical gland removal. The sudden drop in PTH levels leads to a sharp decline in bone resorption and a corresponding increase in calcium and phosphate deposition into bone tissue, often causing significant and prolonged hypocalcemia. Paradoxically, this may also lead to hypophosphatemia, in contrast to the hyperphosphatemia typically observed in hypoparathyroidism in patients with normal renal function (17).

Therefore, timely management before the onset of extensive bone remodeling is critical to preserving both function and quality of life. Nevertheless, this case emphasizes the urgent need to strengthen CKD management programs in developing countries, focusing on the early prevention and treatment of hyperparathyroidism-related complications.

## Conclusion

Tertiary hyperparathyroidism (THPT) is a severe and uncommon complication of secondary hyperparathyroidism in patients with advanced chronic kidney disease, especially those on long-term dialysis or lacking access to kidney transplantation. This case highlights the devastating consequences of uncontrolled THPT, including mineral-bone disorders and widespread systemic deterioration.

In resource-limited settings like Honduras, economic barriers and the strain on healthcare systems restrict access to effective treatments, increasing the risk of progression to severe and often irreversible complications. This case underscores the critical need for more accessible and effective preventive and therapeutic strategies, as well as the development of kidney transplant programs in developing countries. Timely interventions such as parathyroidectomy remain essential for improving the prognosis of THPT patients, though their success largely depends on early diagnosis and the availability of resources (4).

## Data Availability

The original contributions presented in the study are included in the article/supplementary material. Further inquiries can be directed to the corresponding author.
